# Ultrasensitive Luminescent In Vitro Detection for Tumor Markers Based on Inorganic Lanthanide Nano‐Bioprobes

**DOI:** 10.1002/advs.201600197

**Published:** 2016-10-20

**Authors:** Wei Zheng, Shanyong Zhou, Jin Xu, Yongsheng Liu, Ping Huang, Yan Liu, Xueyuan Chen

**Affiliations:** ^1^ CAS Key Laboratory of Design and Assembly of Functional Nanostructuresand State Key Laboratory of Structural ChemistryFujian Institute of Research on the Structure of MatterChinese Academy of SciencesFuzhouFujian350002China

**Keywords:** dissolution‐enhanced luminescence, lanthanides, nano‐bioprobes, tumor markers, ultrasensitive bioassays

## Abstract

Ultrasensitive and accurate detection of tumor markers is of vital importance for the screening or diagnosis of cancers at their early stages and for monitoring cancer relapse after surgical resection. Inorganic lanthanide (Ln^3+^) nanoparticles (NPs), owing to their superior physicochemical characteristics, are regarded as a new generation of luminescent nano‐bioprobes in the field of cancer diagnosis and therapy. In this progress report, a focus is set on our recent efforts on the development of inorganic Ln^3+^‐NPs as efficient luminescent nano‐bioprobes for the ultrasensitive in vitro biodetection of tumor markers, with an emphasis on the dissolution‐enhanced luminescent bioassay (DELBA), an emerging technique recently developed toward practical medical applications.

## 1 Introduction

Cancer is the uncontrolled growth and spread of cells. It affects almost any part of the body and figures among the leading causes of morbidity and mortality worldwide.^1^ Nonetheless, for many cancers, there remains a high chance of cure if they are detected early and treated effectively.^2^ Since the occurrence and development of cancer are always accompanied with the appearance or increase of specific biomolecules in human body fluids (e.g., serum, saliva, and urine), these biomolecules can be exploited as tumor markers. Sensitive and accurate detection or tracing of these biomarkers is essential for early diagnosis or screening of cancer, which offers remarkable opportunities for intervention and will eventually increase the patient's survival and quality of life.^3^ However, it is still a great challenge to trace the low levels of tumor markers in the early stages of cancer. It was reported that ≈75% of lung‐cancer patients were diagnosed at late stages and no effective treatment was available, while more than 70% of patients can survive another 5 years if treated early.^4^

Meanwhile, tumor markers can decrease to a very low level that is beyond the detection capability of current commercial bioassay methods after surgical resection of cancerous tissues, and a series of increase can be an indicator of the recurrence or metastasis of cancers.^5^ Therefore, it is highly demanded to develop ultrasensitive and reliable bioassay methods to trace the ultralow levels of tumor markers for early diagnosis or screening of cancers as well as for evaluating the efficacy of the therapy.

**Figure 1:**
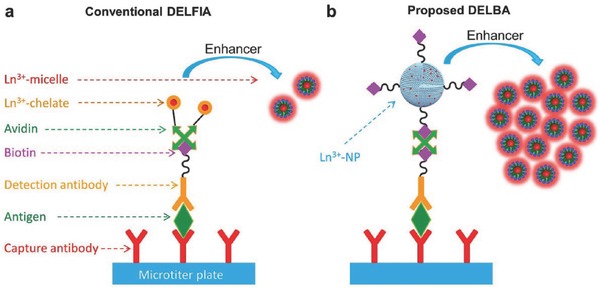
Schematic representation of a) conventional DELFIA based on organic Ln^3+^‐chelates and b) the proposed DELBA based on inorganic Ln^3+^‐NPs. Due to the highly concentrated Ln^3+^ ions in a single NP (DELBA), a myriad of Ln^3+^ ions can be released from the NPs and transformed into highly luminescent Ln^3+^‐micelles after addition of the enhancer solution, which yields a significantly amplified TRPL signal and thereby a much higher detection sensitivity than conventional DELFIA.

In the past decades, diverse immunoassay methods including radioimmunoassay (RIA), enzyme linked immunosorbent assay (ELISA), chemiluminescence immunoassay (CLIA), and time‐resolved fluoroimmunoassay (TRFIA) have been developed for the detection of tumor markers in clinical diagnosis.^6^ RIA and ELISA, owing to the deficiencies of radioactive contamination, instability of enzyme substrate and low sensitivity, were gradually replaced by CLIA and TRFIA in current market of cancer diagnosis. Specifically, dissociation‐enhanced lanthanide fluoroimmunoassay (DELFIA), as a representative of TRFIA, is recognized as one of the most sensitive commercial bioassay methods.^7^ The technique of DELFIA employs a non‐luminescent lanthanide (Ln^3+^)‐chelate to label the analyte, which is subsequently transformed into a highly luminescent Ln^3+^‐micelle through reaction with a weakly acidic photoluminescence (PL) enhancer solution (**Figure** **1**a).[[qv: 7a]] As a result, the PL of the labels can be enormously enhanced via the “antenna effect” from the enhancer solution. Moreover, by taking advantage of the time‐resolved (TR) technique, the background noise from scattered light and other short‐lived biological autofluorescence can be completely eliminated in DELFIA, thus providing a remarkably higher sensitivity than conventional steady‐state fluorescence immunoassays.[[qv: 6b]] However, the use of molecular probes like Ln^3+^‐chelates as biolabels suffers from a series of disadvantages, such as a low labeling ratio of Ln^3+^ per biomolecule (up to 10–30), poor chemical stability and high cost of Ln^3+^‐chelating agents, which strongly restricts the sensitivity of the assay and its widespread applications.[[qv: 6d]]

Inorganic lanthanide nanoparticles (NPs), owing to their superior physicochemical properties such as high photochemical stability, numerous luminescent centers in a single NP‐label, excellent flexibility for bioconjugation, non‐toxicity and low cost, are emerging as a new class of luminescent nanoprobes and as an alternative to conventional molecular probes for versatile bioapplications.^8^ With the rapid development of nanotechnology and biotechnology in recent years, many significant advancements have been achieved for Ln^3+^‐NPs, including their controlled synthesis, functional design, and their promising bioapplications in the field of cancer theranostics.^9^ Despite the achievements, Ln^3+^‐NPs yet suffer from low‐to‐medium brightness as compared to conventional fluorescence bioprobes such as organic dyes, quantum dots and Ln^3+^‐chelates, which results in the poor sensitivity in bioassays.^10^ The intrinsic origin of the low PL efficiency of Ln^3+^‐NPs lies in the small extinction coefficient of Ln^3+^ (on the order of 1 M^–1^ cm^–2^) due to the parity‐forbidden nature of the intra‐4f^N^ transitions.^11^

To overcome these concerns, we have recently developed a novel bioassay method, namely, dissolution‐enhanced luminescent bioassay (DELBA), for the ultrasensitive in vitro detection of tumor markers,^12^ which integrates hybrid advantages of inorganic Ln^3+^‐NPs and organic Ln^3+^‐chelates. The proposed DELBA follows the protocol of commercial DELFIA except that Ln^3+^‐NPs instead of Ln^3+^‐chelates are employed in the labeling process (Figure. 1b). As a result, the labeling ratio of Ln^3+^ per biomolecule (up to tens of thousands of Ln^3+^ depending on the size of the NPs) is enormously enhanced due to the numerous Ln^3+^ ions in a single NP. Meanwhile, through the sensitization of specific PL enhancer solution, the issue of the low PL efficiency of Ln^3+^‐NPs is solved simultaneously. As such, in the assay process, a large quantity of Ln^3+^ ions can be released from the NPs and transformed into highly luminescent Ln^3+^‐micelles via reaction with the enhancer solution, which yields a significantly amplified TRPL signal and thereby a much higher detection sensitivity than conventional DELFIA.

In this progress report, we focus on our recent efforts on the development of DELBA for the ultrasensitive in vitro detection of tumor markers. The general protocols for the fabrication of inorganic Ln^3+^‐nanoprobes including the chemical synthesis, surface functionalization, and bioconjugation are overviewed in Section 2. In Section 3, we discuss the mechanism on the dissolution‐enhanced luminescence of Ln^3+^‐nanoprobes in the PL enhancer solution. Some important parameters that may influence the performance of the nanoprobes in DELBA are specifically outlined. Section 6 highlights the key applications of DELBA for the ultrasensitive in vitro detection of several important tumor markers including carcinoembryonic antigen (CEA), prostate specific antigen (PSA), and alpha‐fetoprotein (AFP). Prospects and future efforts towards the commercialization of this new technique are also envisioned.

## 2 General Protocols for the Fabrication of Inorganic Lanthanide Nanoprobes

For applications in DELBA, the nanoprobes should be monodispersed, small sizes, easy of dissolution in the enhancer solution, and capable of specific recognition of targeted biomolecules. In this section, we provide a brief overview of the general routes to the preparation of inorganic Ln^3+^‐nanoprobes especially for DELBA, including their controlled synthesis, surface modification and bioconjugation.

### 2.1 Controlled Synthesis

Currently, there are three prevailing methods for the controlled synthesis of inorganic Ln^3+^‐NPs, namely, thermal decomposition, high‐temperature coprecipitation, and hydro(solvo)‐thermal.^13^ In these methods, long‐chain organic surfactants (hydrophilic or lipophilic) are often used to control the nucleation and growth of the nanocrystals. The utilization of hydrophilic surfactants such as ethylenediaminetetraacetic acid (EDTA), polyethylenimine (PEI), polyacrylic acid (PAA), polyethylene glycol (PEG), and 2‐aminoethyl dihydrogen phosphate (AEP) enables one‐step synthesis of hydrophilic and biocompatible NPs, but usually yields NPs with poor uniformity and monodispersity.^14^ Instead, the lipophilic surfactants such as oleic acid (OA) and oleylamine (OM) along with other high boiling organic solvents like 1‐octadecene (ODE) are favorable for the synthesis of high‐quality NPs with a narrow size distribution, good crystallinity, defined core/shell nanostructures, and excellent optical properties.

**Figure 2:**
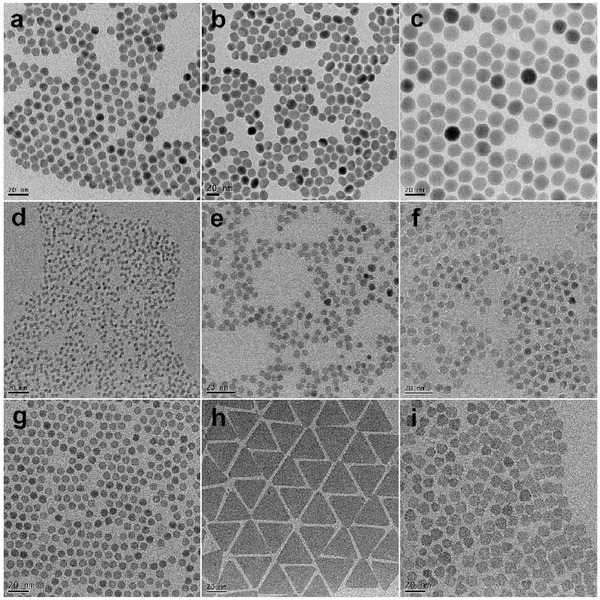
TEM images of typical Ln^3+^‐NPs synthesized using a–c) high‐temperature co‐precipitation and d–i) thermal decomposition methods in the presence of OA/OM as surfactants in our lab. a) β‐NaEuF_4_, b) β‐NaSmF_4_, c) β‐NaGdF_4_:Eu^3+^, d) cubic LaOF, e) orthorhombic Lu_6_O_5_F_8_, f) tetragonal YOF, g) circular Eu_2_O_3_ nanoplates, h) triangular Eu_2_O_3_ nanoplates, and i) Sm_2_O_3_ nanocubes (scale bars = 20 nm).

Through thermal decomposition and high‐temperature coprecipitation methods in the presence of OA, OM and ODE, we have synthesized a number of high‐quality lanthanide fluoride, oxyfluoride and oxide NPs with controlled crystalline phases, morphologies, sizes, and optical properties. For example, based on a high‐temperature coprecipitation method, a series of AREF_4_ (A = Li, Na, K; RE = Eu, Sm, Gd, Y, Lu, et al.) NPs with different crystalline phases and particle sizes ranging from several to hundreds of nm were synthesized (**Figures** **2**a–c).^15^ Through a modified thermal decomposition method, ultrasmall (<5 nm) cubic LaOF, orthorhombic Lu_6_O_5_F_8_, and tetragonal YOF NPs were fabricated (Figures 2d–f).^16^ Similarly, by a thermal decomposition method with LiOH as a mineralizer, RE_2_O_3_ nanoplates with circular, tripodal and triangular morphologies were obtained (Figures 2g–i). Such controlled syntheses of Ln^3+^‐NPs are favorable for screening ideal nanoprobe candidates for DELBA, since NPs with different compositions, sizes and morphologies may contain different amount of luminescent ions and exhibit distinct dissolution behavior in the PL enhancer solution.

### 2.2 Surface Modification and Bioconjugation

**Figure 3:**
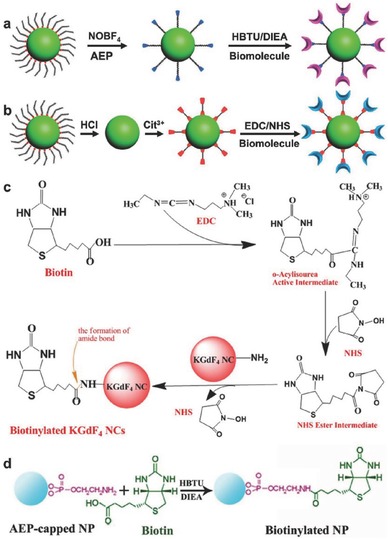
Schematic illustration of two representative protocols for the surface functionalization of inorganic Ln^3+^‐NPs: a) ligand exchange and b) ligand‐free synthesis. Schematic illustration of c) the bioconjugation of KGdF_4_ NPs with biotin through a standard EDC/NHS bioconjugation protocol and d) biotinylation of AEP‐capped ZrO_2_ NPs in DMF by the use of HBTU and DIEA as the coupling reagents. Reproduced with permission.[[qv: 22,19a]] Copyright 2012, American Chemical Society.

Because of the lipophilic ligands such as OA or OM anchored to the surface, the as‐synthesized Ln^3+^‐NPs are hydrophobic and incompatible with biological systems. Therefore, surface modification is usually demanded to transfer the hydrophobic NPs into hydrophilic ones with appropriate functional groups (e.g., amino and carboxy) for subsequent bioconjugation (**Figures** **3**a and 3b).^17^ For this purpose, a number of surface modification strategies have been established, such as ligand exchange, ligand oxidation, ligand‐free synthesis, ligand attraction, electrostatic layer‐by‐layer assembly, and surface silanization.^18^

Through ligand exchange from the as‐prepared hydrophobic NPs by using AEP as surfactants, we synthesized amine‐functionalized CaF_2_:Ln^3+^ and ZrO_2_:Ln^3+^ NPs for the conjugation of amino terminal fragment (ATF) of urokinase plasminogen activator (uPA).^19^ The ATP‐capped NPs were able to specifically recognize uPA receptor (uPAR, an important tumor marker) and were explored as sensitive TRPL and upconverting luminescence (UCL) nanoprobes for the in vitro detection of uPAR and for uPAR‐targeted cancer cell imaging. Specifically, through a simple acid‐washing treatment, the capping ligands on the surface of the hydrophobic NPs can be removed, yielding water‐soluble and ligand‐free NPs. As a consequence, positively charged Ln^3+^ ions are exposed on the surface of the ligand‐free NPs, thus enabling the NPs for direct conjugation in various buffer solutions with hydrophilic and biocompatible molecules, through either electrostatic attraction or the strong chelation of Ln^3+^.[[qv: 18f]] For example, via electrostatic attraction, avidin was successfully and stably attached to the surface of ligand‐free LiLuF_4_:Ln^3+^ NPs. The avidin‐functionalized LiLuF_4_:Ln^3+^ NPs were further explored as sensitive UCL nanoprobes for the detection of β subunit of human chorionic gonadotropin (β‐hCG, an important pregnancy indicator and tumor marker) with a limit of detection (LOD) of ≈3.8 ng mL^–1^, comparable to the β‐hCG level in the serum of normal humans.[[qv: 15g]] Similarly, biotin was conjugated to the surface of NaEuF_4_, Sr_2_YF_7_:Ln^3+^, and NaGdF_4_:Ln^3+^@NaEuF_4_ core/shell NPs through the strong chelation of Ln^3+^ in a ligand‐free synthesis.^12^, ^20^ The biotinylated NPs were able to serve as general and multifunctional nano‐bioprobes in DELBA for the ultrasensitive in vitro detection of diverse tumor markers, as will be outlined in Section 6.

Besides the direct bioconjugation through ligand‐free synthesis, chemical bonding between the functional groups on the surface of inorganic Ln^3+^‐NPs (e.g., maleimide, thiol, carboxylic, aldehyde, and amine) and those of the biomolecules are most prevalent for the bioconjugation of Ln^3+^‐NPs.^21^ Cross‐linking reagents such as ethyl(dimethylaminopropyl) carbodiimide (EDC) and N‐hydroxysuccinimide (NHS), *o*‐benzotriazole‐*N,N,N′,N′*‐tetramethyluronium‐hexafluoro‐phosphate (HBTU) and *N,N*‐diisopropylethy (DIEA), dicyclohexylcarbodiimide (DCC) and 1‐hydroxybenzotriazole (HOBT) have been employed to activate the functional groups of biomolecules (Figures 3c and 3d). For instance, avidin was conjugated to the surface of ultrasmall citrate‐modified Lu_6_O_5_F_8_:Eu^3+^ NPs following a standard EDC/NHS bioconjugation protocol.^16^ Biotin and ATF of uPA were coupled with amine‐functionalized CaF_2_:Ln^3+^ and ZrO_2_:Ln^3+^ NPs in *N,N*‐dimethylformamide (DMF) by using HBTU/DIEA as cross‐linking reagents.^19^ Note that the bioactivity of the conjugating biomolecules as well as the dispersability of the NPs may decrease during the bioconjugation, therefore the species and the amounts of the cross‐linking reagents should be carefully selected. Meanwhile, to avoid non‐specific binding in subsequent bioassays, it is highly desirable to block the residual binding sites on the surface of the NPs. For this purpose, bovine serum albumin (BSA) and human serum albumin (HSA) are often used as the blocking reagents.

## 3 Dissolution‐Enhanced PL of Inorganic Ln^3+^‐NPs

Trivalent lanthanide ions are excellent luminescent centers in inorganic NPs owing to their abundant electronic energy levels within [Xe]4f^N^ (N = 0−14) electronic configurations. However, because the intra‐4f^N^ transitions of Ln^3+^ are parity forbidden, Ln^3+^ ions generally possess a small absorption cross‐section with a narrow absorption band, which results in a low PL efficiency upon direct excitation of Ln^3+^. To address this issue, it is a smart choice to introduce an antenna that can boost the absorption efficiency and sensitize the Ln^3+^ luminescence. Optical entities of allowed transitions such as Ce^3+^, Bi^3+^, [VO_4_]^3−^, Ln^3+^–O^2−^ charge‐transfer states, exciton recombination of semiconductors as well as the singlet−triplet transitions of organic complexes, are ideal antenna candidates for Ln^3+^ luminescence.[[qv: 10c,23]] In this regard, the aromatic chelating ligands like 2‐naphthoyltrifluoroacetone (β‐NTA), thienoyltrifluoroacetone (TTA) and pivaloyltrifluoroacetone (PTA) that are well‐known antennas in Ln^3+^‐chelates,[[qv: 23b]] may be employed to sensitize the luminescence of Ln^3+^‐NPs.

**Figure 4:**
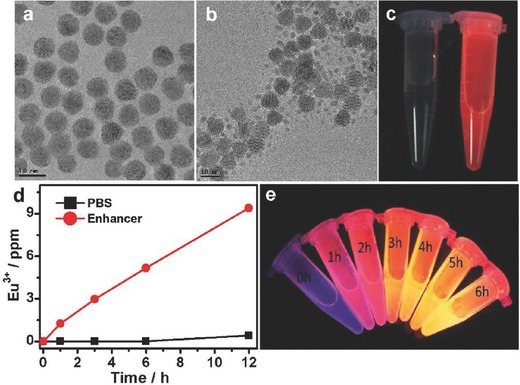
TEM images of a) ligand‐free NaEuF_4_ NPs and b) their addition to the enhancer solution (scale bars = 10 nm). c) Images showing the PL of ligand‐free NaEuF_4_ NPs in PBS (left) and the enhancer solution (right) under UV lamp illumination at λ = 300 nm. d) The concentration of Eu^3+^ ions in the dialysate of ligand‐free NaEuF_4_ NPs after 12 hours dialysis in the enhancer solution and PBS, respectively. e) Images of the dialysate for ligand‐free NaEuF_4_ NPs dialyzed in the enhancer solution under UV lamp illumination at λ = 300 nm, retrieved at different time intervals (1–6 h). Reproduced with permission.^12^

**Figure 5:**
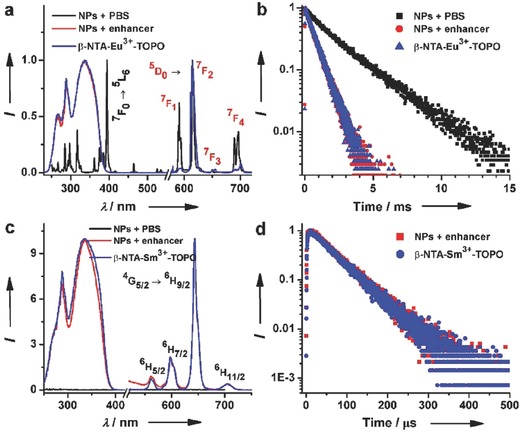
a) Normalized PL excitation (left) and emission spectra (right) of ligand‐free NaEuF_4_ NPs in PBS and the enhancer solution, and β‐NTA–Eu^3+^–TOPO ternary complex, respectively. b) The corresponding PL decays from ^5^D_0_ by monitoring the Eu^3+^ emission at 614 nm. c) Normalized PL excitation (left) and emission spectra (right) of ligand‐free NaSmF_4_ NPs in PBS and the enhancer solution, and β‐NTA–Sm^3+^–TOPO ternary complex, respectively. d) The corresponding PL decays from ^4^G_5/2_ by monitoring the Sm^3+^ emission at 643 nm. Reproduced with permission.^12^

To search for the effective sensitizer of Ln^3+^‐NPs, we discovered that the PL of Eu^3+^ (or Sm^3+^) NPs can be significantly enhanced by a simple addition of the NPs into the enhancer solution (pH 2.3) of commercial DELFIA consisting of β‐NTA, TOPO and Triton X‐100, regardless of the phases, sizes and morphologies of the NPs.^12^ To unveil this interesting phenomenon, we added the ligand‐free NaEuF_4_ NPs into the enhancer solution containing β‐NTA, which has a very high affinity for Eu^3+^ ions (log *k* = 8.8).[[qv: 23b]] Transmission electron microscope (TEM) images (**Figures** **4**a and 4b) showed that a large fraction of the original NPs became smaller in the enhancer solution, indicative of a chemical dissolution of the NPs. Besides, an unprecedented amplification (10^6^ times) of the PL was observed (Figure 4c). The dynamic dissolution process of the NPs was further corroborated by the dialysis experiments that interpret a gradual release of Eu^3+^ from the NPs (Figures 4d and 4e). By means of high‐resolution optical spectroscopy, the enhanced PL was revealed to originate from β‐NTA–Eu^3+^–TOPO complex instead of the original NaEuF_4_ NPs. It was found that the PL pattern and the PL lifetime of the NPs in the enhancer solution were drastically different from those of the NPs in phosphate‐buffered saline (phosphate buffer saline (PBS), pH 7.4), but exactly identical to those of β‐NTA–Eu^3+^–TOPO complex (**Figures** **5**a and 5b). Similar results were also found in Sm^3+^‐NPs such as NaSmF_4_ and Sm_2_O_3_ (Figures 5c and 5d). The dissolution mechanism of the chemically stable NaEuF_4_ NPs can be briefly depicted as the following dynamic process: upon addition of the enhancer solution, trace amount of Eu^3+^ ions detached from ligand‐free NaEuF_4_ NPs react instantly with β‐NTA followed by the formation of β‐NTA‐Eu^3+^‐TOPO; such a reaction would promote the release of Eu^3+^ from the NPs at the expense of β‐NTA in the enhancer solution, until it reaches a chemical equilibrium. Because the ligand of β‐NTA has a very high molar extinction coefficient (5.82 × 10^4^ cm^–1^ M^–1^) at ≈340 nm,[[qv: 23b]] the complex newly formed can efficiently harvest the UV light and transfer the excitation energy to the coordinated Eu^3+^, thus resulted in the enormously enhanced PL of the dissolved NPs relative to their original counterparts dispersed in PBS.

**Figure 6:**
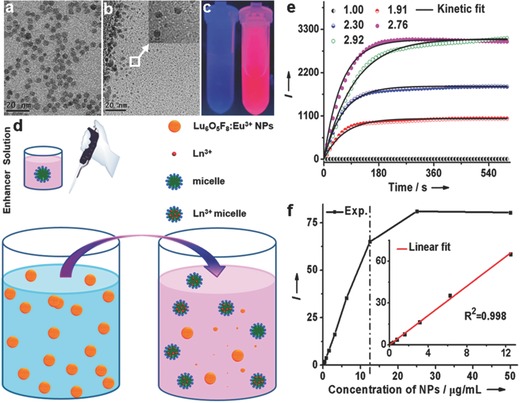
TEM images of a) ligand‐free Lu_6_O_5_F_8_:Eu^3+^ NPs and b) their addition to the enhancer solution. The inset shows the enlarged HRTEM image of the NPs indicated by a white square. c) Photograph showing the PL of ligand‐free Lu_6_O_5_F_8_:Eu^3+^ NPs in PBS (left) and the enhancer solution (right) under UV lamp illustration at λ = 300 nm. d) Schematic representation for the dissolution mechanism of Lu_6_O_5_F_8_:Eu^3+^ NPs. e) Kinetic fitting of the dissolution‐enhanced PL signal of ligand‐free Lu_6_O_5_F_8_:Eu^3+^ NPs (50 μg mL^–1^) dissolved in the enhancer solution at pH = 1.00, 1.91, 2.30, 2.76 and 2.92. f) Concentration‐dependent dissolution‐enhanced PL signal of ligand‐free Lu_6_O_5_F_8_:Eu^3+^ NPs dissolved in the enhancer solution. Inset: linear range of the PL signal versus the NP concentration (0–12.5 μg mL^–1^). e‐f) Each data point represents the mean of triplicate experiments. Reproduced with permission.^16^ Copyright 2016, Royal Society of Chemistry.

In addition to fluorides, we also extended the strategy of the dissolution‐enhanced PL to other kinds of Ln^3+^‐NPs such as oxyfluorides and oxides. In comparison with fluorides, lanthanide oxyfluorides and oxides are more liable to be dissolved in the acidic enhancer solution, since the Ln—O bond can be more easily loosened than the Ln—F bond via the protonation reaction with H^+^.^24^ For example, a large quantity of tiny NPs (<3 nm) can be observed in TEM images immediately upon addition of Lu_6_O_5_F_8_:Eu^3+^ NPs into the enhancer solution (**Figures** **6**a and 6b), indicative of a faster dissolution response than that of NaEuF_4_ NPs.^16^ The dissolved NPs exhibited a strong deep pink emission under an UV lamp illumination, in stark contrast to the NPs dispersed in PBS (Figure 6c). By means of high‐resolution optical spectroscopy, the enhanced PL was unambiguously attributed to the Eu^3+^‐complexes instead of the original Lu_6_O_5_F_8_:Eu^3+^ NPs, similar to the case in NaEuF_4_. Since the Eu^3+^‐complexes are formed through the coordination reaction between the chelating ligands in the enhancer solution and the Eu^3+^ ions releasing from the NPs (Figures 6d), the intensity of the dissolution‐enhanced PL of the NPs depends critically on the molar concentration of Eu^3+^ and the dissolution behavior of the NPs, which relies heavily on the pH value of the enhancer solution. The optimal Eu^3+^ concentration in Lu_6_O_5_F_8_ NPs was determined to be 40 %, due to the fact that the inert Lu^3+^‐complex may alleviate the concentration quenching of Eu^3+^ and enhance the luminescence of Eu^3+^‐complex through a co‐fluorescence effect.[[qv: 6d]] By simulating the dissolution process based on a set of kinetic equations, the pH value of the enhancer solution was optimized to be 2.76 for Lu_6_O_5_F_8_:Eu^3+^ NPs. Under the optimum conditions, the dissolution‐enhanced PL of the NPs was observed with a fast response within 5 minutes and a broad linear dynamic range versus the NP concentration from 0 to 12.5 μg mL^–1^ (Figures 6e and 6f), which are of key importance for quantitative fluorescence biolabeling.

It is worthy of emphasizing that, the structure and crystal‐field strength of the crystal lattice and the localized electronic structures of Ln^3+^ that normally influence significantly the PL efficiency of inorganic Ln^3+^‐NPs, are no longer the decisive factors on their dissolution‐enhanced PL, since the NPs are dissolved and transformed into Ln^3+^‐micelles in the enhancer solution. Instead, it is the chelating ligands of the enhancer solution, the molar concentration of Ln^3+^, and the dissolution behavior of the NPs that dictate the dissolution‐enhanced PL behavior of the NPs. Meanwhile, it is expected that such PL enhancement is universal and can be extended to other Ln^3+^‐chelating systems, for example, Tb^3+^/Dy^3+^‐NPs and the enhancer solutions containing PTA. More importantly, in view of the numerous Ln^3+^ ions in a single NP, the dissolution‐enhanced PL of inorganic Ln^3+^‐NPs can be treated as an ideal signal amplification strategy and as an alternative to conventional DELFIA for the ultrasensitive detection of tumor markers.

## 4 Ultrasensitive In Vitro Detection of Tumor Markers

Based on the superior optical properties of inorganic Ln^3+^‐nanoprobes such as the long‐lived downshifting luminescence (DSL) and the NIR‐triggered anti‐Stokes UCL, a series of novel bioassay methods such as the heterogeneous TRPL and UCL assays, the homogeneous TR Förster resonance energy transfer (FRET) and UC‐FRET assays have been developed for the detection of various tumor markers.[[qv: 8f,19b,25]] However, these bioassays, despite the advantage of background‐free signal, are strongly restricted by the low PL efficiency of Ln^3+^‐nanoprobes. Recently, by taking advantages of the drastically enhanced PL of inorganic Ln^3+^‐nanoprobes in the enhancer solution, we developed a unique bioassay method, namely, DELBA, for the ultrasensitive in vitro detection of several important tumor markers such as CEA, PSA and AFP, with LODs several orders of magnitude improvement on those of either commercial TRFIA or the assays utilizing the PL of the original Ln^3+^‐nanoprobes (**Table** **1**), as exemplified in the following subsections.

**Table 1:** Representative assays of tumor markers utilizing Ln^3+^‐based luminescent bioprobes

Bioprobes	Size (nm)	Bioconjugation	Analyte	LOD (pg mL^–1^)	Assay format	Ref.
CaF_2_:Ce,Tb	≈3.8	ATF	suPAR	≈2.0 × 10^4^	TR‐FRET	[[qv: 19b]]
Tb‐BPTA	—	Strepavidin	AFP	42	TRFIA	26
Tb‐BPTA	—	Strepavidin	CEA	70	TRFIA	26
Eu‐NTA‐polystyrene NPs	≈41	Antibody	PSA	1.6	TRFIA	27
NaEuF_4_	8.5	Biotin	CEA	0.1	DELBA	12
NaSmF_4_	15	Biotin	CEA	1.2	DELBA	12
Lu_6_O_5_F_8_:Eu	4.8	Avidin	PSA	0.52	DELBA	16
NaGdF_4_:Ln^3+^ CSS	23	Biotin	AFP	60	DELBA	[[qv: 20b]]
NaGdF_4_:Ln^3+^ CSS	23	Biotin	AFP	20	UCL	[[qv: 20b]]
NaYF_4_:Yb,Er	Bulk	Strepavidin	PSA	0.53	UCL	[[qv: 25f]]
LiLuF_4_:Yb,Er@LiLuF_4_	50	Avidin	β‐hCG	3.8 × 10^3^	UCL	[[qv: 15g]]
NaYF_4_:Yb,Er @ NaYF_4_	35	Antibody	CEA	50	UC‐FRET	[[qv: 25h]]
NaYF_4_:Yb,Er,Mn	≈42	Antibody	PSA	113	UC‐FRET	[[qv: 25g]]

### 4.1 CEA

CEA is normally produced in gastrointestinal tissue during fetal development, and present at low levels (<5 ng mL^–1^) in healthy adults.^28^ Elevated levels of CEA in the serum of individuals may be related with colorectal cancer, gastric cancer, pancreatic cancer, lung cancer, breast cancer, and medullary thyroid cancer.^29^ CEA is recognized as a broad‐spectrum tumor marker and is a prerequisite test item in hospitals for cancer screening. CEA measurement is especially useful to monitor cancer treatment, to identify recurrences after surgical resection, to localize cancer spread, for staging or for prognosis.^30^ After surgical resection of cancerous tissues, the concentration of CEA usually decreases to a very low level and a series of increase in CEA level can be an indicator of the recurrence or metastasis of cancers.^31^ Therefore, it is of critical importance to detect ultra‐low level of CEA for the screening and monitoring of cancers.

**Figure 7:**
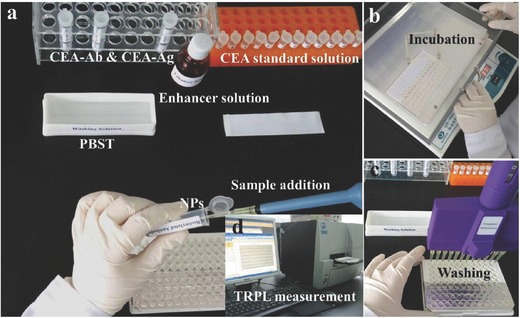
Photographic representation illustrating the operation procedures for the assay of CEA: a) sample addition, b) incubation, c) washing, and d) TRPL measurement.

Recently, based on the new technique of DELBA, we realized the ultrasensitive detection of CEA in human serum samples.^12^ NaEuF_4_ NPs with a mean size of ≈8.5 nm (≈4300 Eu^3+^ ions per NP) were synthesized and employed as the DELBA nanoprobes after conjugation with biotin. The biotinylated nanoprobes can indirectly capture the biotinylated biomolecules through the bridging of avidin.^32^ By virtue of the specific recognition of the anti‐CEA antibody with CEA, a sandwich‐type DELBA was built with the labeling of the biotinylated NaEuF_4_ nanoprobes. A typical process of DELBA for CEA assay is briefly illustrated as follows (**Figure** **7**): First, anti‐CEA antibody was coated on a 96‐well microplate via incubation; human CEA standard solution was then added to bind with the antibody; thereafter, biotinylated anti‐CEA antibody was added to conjugate with CEA, forming a double antibody sandwiched CEA structure; after that, avidin was added to bridge the biotinylated antibody and the biotinylated NaEuF_4_ NPs through biotin‐avidin‐biotin interactions, thus realizing the labeling of the nanoprobes on the sandwiched CEA; finally, the enhancer solution was added to dissolve the NPs and generate the dissolution‐enhanced PL, and the plate was subjected to TRPL measurement. CEA can be then quantified by the dissolution‐enhanced TRPL of the nanoprobes. Note that each step involves incubation and washing operations, and different buffer solutions were used throughout the labelling process. Experimental details can be found in the supporting information of Reference.^12^

**Figure 8:**
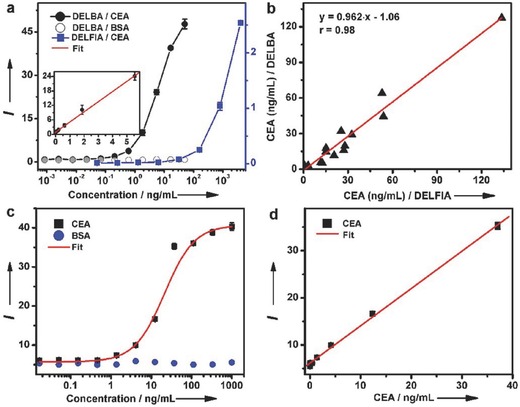
a) Calibration curves for the CEA assay using DELBA (based on NaEuF_4_ NPs) and the commercial DELFIA kit (based on the Eu^3+^–DTTA complex). Right‐hand axis refers to DELFIA/CEA square data points. Gray circles indicate overlapped black and white circular data points. Inset: the linear range of the calibration curve for the CEA assay by DELBA. b) Correlation between DELBA and commercial DELFIA for the detection of CEA in 20 human serum samples. c) Calibration curve and d) its linear dynamic range for CEA assay using DELBA (based on NaSmF_4_ NPs). Each data point represents the mean (±standard deviation) of three independent experiments. Reproduced with permission.^12^

Based on the intense dissolution‐enhanced PL of NaEuF_4_ NPs, we realized the ultrasensitive CEA detection with a linear dynamic range of 0.1 pg mL^–1^–10 ng mL^–1^ and a LOD of 0.1 pg mL^–1^ (0.5 fM), which is almost 1000‐fold improvement relative to that of commercial DELFIA (≈90 pg mL^–1^) (**Figure** **8**a).^26^ The CEA levels of 20 human serum samples determined by DELBA were compared with those measured independently using a commercial DELFIA kit, yielding a good agreement with a correlation coefficient of ≈0.98 (Figure 8b). The coefficient of variations (CVs) of the assays were below 8% and the recoveries were in the range of 95–105%, thus confirming the excellent accuracy and precision of DELBA (**Table** **2**). Furthermore, the DELBA system was also extended to other Ln^3+^‐nanoprobes. For example, by employing the biotinylated NaSmF_4_ and Sr_2_YF_7_:Eu^3+^ NPs as the DELBA nanoprobes, we achieved LODs of ≈6.0 fM and ≈95 pM for CEA, respectively (Figures 8c and 8d).[[qv: 20a]] More recently, benefiting from the high sensitivity of DELBA, we realized the detection of CEA in saliva, in which the CEA level is much lower than in serum.^33^ The salivary diagnostics of tumor markers provides a more convenient and non‐invasive sampling strategy than conventional serum‐based assays, therefore is highly promising for cancer screening and monitoring in future family and community medical service.

**Table 2:** Assay precision and analytical recovery of CEA, PSA and AFP added to serum samples

Tumor Markers	Added (ng mL^–1^)	Found (ng mL^–1^)	CV (%) (n = 4)a)	Recovery (%)
CEA	Sample 1	6.91	7.15	——
	0.50	7.40	6.87	98.10
	10.7	17.1	1.22	95.10
	56.0	65.5	4.40	104.6
	Sample 2	33.4	4.63	——
	10.7	44.1	2.99	100.3
	56.0	91.9	3.38	104.6
PSA	Sample 1	2.99	3.57	——
	2.50	5.65	3.98	106.51
	3.00	5.78	2.13	93.24
	12.00	15.87	1.06	107.36
	Sample 2	1.83	5.09	——
	1.00	2.81	7.36	98.17
	7.00	9.01	2.88	102.59
	28.00	27.76	2.03	92.60
AFP	Sample 1	5.1	2.36	——
	0.5	5.6	3.28	108.1
	10.7	16.2	4.42	103.7
	56.0	62.4	4.40	102.3

^a)^Intra‐assay using four different wells in a plate.

### 4.2 PSA

PSA is a member of the kallikrein‐related peptidase family and is secreted by the epithelial cells of the prostate gland.^34^ The serum PSA level in men with healthy prostates is very low, and an elevated PSA level can be indicative of prostate cancer (PCa) or other prostate disorders.^35^ Especially, a PSA level of >4 ng mL^–1^ is regarded as an indicator of suspicion of PCa.^36^ But after radical prostatectomy, the serum PSA usually decreases to an undetectable level (<5 pg mL^–1^) within a few week, and a subsequent rise with a value above 0.2 ng mL^–1^ is generally considered as an evidence of recurrent PCa.^37^ Therefore, it is significant to detect or monitor the low levels of PSA in a wide range from ≈1 pg mL^–1^ to >4 ng mL^–1^, which is crucial for the diagnosis and therapy of PCa.

**Figure 9:**
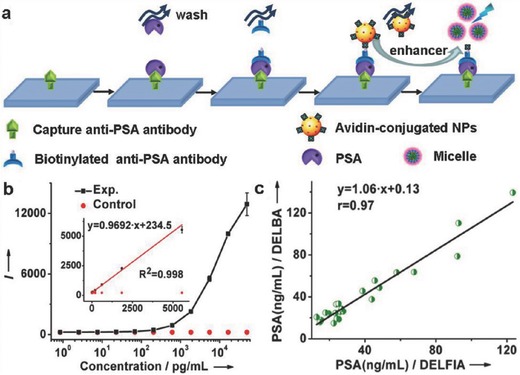
a) The process and principle of a heterogeneous assay for the detection of PSA. b) Calibration curve for the PSA assay based on Lu_6_O_5_F_8_:Eu^3+^ NPs. Inset: the linear range (8.5 × 10^–4^ to 5.6 ng·mL^–1^) of the calibration curve with the regression equation of y = 0.9692x+ 234.5 (*R* ^2^ = 0.998). c) Correlation between the NP‐based assay and commercial DELFIA kit for the detection of PSA in 23 patient serum samples. b and c) Each data point represents the mean (±standard deviation) of triplicate experiments. Reproduced with permission.^16^ Copyright 2016, Royal Society of Chemistry.

To realize the ultrasensitive PSA detection, we have recently developed ultrasmall Lu_6_O_5_F_8_:Eu^3+^ nanoprobes in a sandwich‐type DELBA through conjugation with avidin.^16^ As illustrated in **Figure** **9**a, PSA was sandwiched between the antibody and the biotinylated antibody, and further labeled with the avidin‐functionalized Lu_6_O_5_F_8_:Eu^3+^ nanoprobes via biotin/avidin interaction. After addition of the enhancer solution, PSA was quantified by measuring the dissolution‐enhanced TRPL signal of the nanoprobes on a microplate reader. The LOD of the assay was determined to be 0.52 pg mL^–1^ (15.2 fM) (Figure 9b), a value of an almost 200‐fold improvement relative to that of commercial DELFIA (0.1 ng mL^–1^), which is the lowest among the luminescent bioassays for PSA ever reported.^38^ Such a low LOD is attributed to the high molar density of Eu^3+^ ions in a single oxyfluoride NP and the high specificity of the assay, as confirmed by the controlled experiments utilizing noncognate proteins like BSA, HSA, CEA, AFP and β‐hCG. More importantly, the assay exhibits an excellently wide linear dynamic range, namely, 8.5 × 10^–4^ to 5.6 ng mL^–1^ (*R* ^2^ = 0.998), thus fulfiling the critical requirement for tracing PSA levels in both early diagnosis of PCa and monitoring PCa relapse after prostatectomy.

To verify the applicability of Lu_6_O_5_F_8_:Eu^3+^ nanoprobes in practical bioassays, we compared the PSA levels of 23 human serum samples derived from DELBA with those detected independently using a commercial DELFIA kit, and a good agreement was acquired with a correlation coefficient of 0.97 (Figure 9c). Other parameters including the CVs (<8%) and the recoveries (in the range of 92–108%) of the assays further confirmed the reliability and practicability of the nanoprobes for PSA assay (Table 2). Furthermore, by taking advantages of the single‐band red UCL and the high mass density of the Lu_6_O_5_F_8_:Yb,Er nanoprobes, we demonstrated proof‐of‐concept UCL/CT dual‐mode bioimaging of cancer cells, thus revealing the great potential of Lu_6_O_5_F_8_:Ln^3+^ NPs as multifunctional nano‐bioprobes in cancer theranostics.

### 4.3 AFP

AFP is a major plasma protein produced by the yolk sac and the liver during fetal development.^39^ The serum AFP level is normally less than 40 ng mL^–1^ and a level above 200 ng mL^–1^ in adults can be an indicator of hepatocellular carcinoma (HCC), germ cell tumors and liver cancer.^40^ Primary HCC leads the second cause of cancer death worldwide.^41^ The serum AFP levels for primary HCC patients often increase under conditions such as periods of rapid liver cancer cell growth, relapse and metastasis.^42^ For early stage diagnosis, an AFP level of >15 ng mL^–1^ is considered to be an reliable indicator of suspicion of primary HCC, while for monitoring the HCC relapse, the AFP level in the serum usually decreases to <20 ng mL^–1^ after the resection of HCC within the first 2 months, and a serial increase indicates the recurrence or metastasis of HCC.^43^

**Figure 10:**
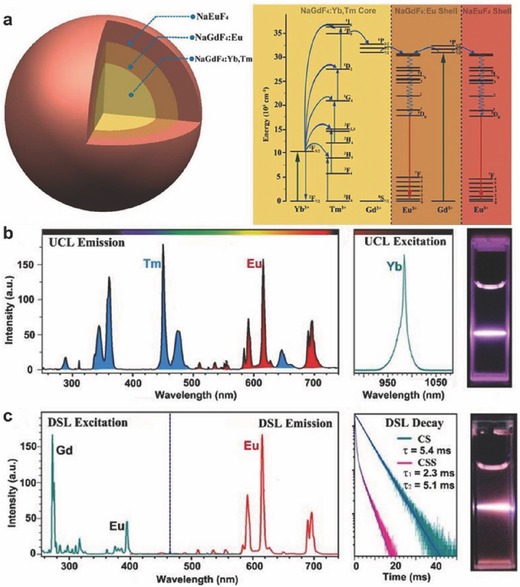
a) Schematic design for the CSS NP comprising NaGdF_4_:Yb/Tm core, NaGdF_4_:Eu and NaEuF_4_ shells to produce intense UCL and DSL of Eu^3+^ (left). Proposed energy transfer mechanisms in the CSS NPs (right). b) UCL emission and excitation spectra showing the Yb^3+^ sensitized UCL of Eu^3+^ (red) and Tm^3+^ (blue) in the as‐synthesized CSS NPs, and the corresponding UCL photograph for the as‐synthesized CSS NPs dispersed in cyclohexane under NIR laser irradiation at 980 nm with a power density of 10 W cm^–2^. c) DSL excitation and emission spectra showing the host Gd^3+^‐sensitized DSL of Eu^3+^, DSL decays from ^5^D_0_ of Eu^3+^ in the CS and CSS NPs by monitoring the Eu^3+^ emission at 615 nm upon UV excitation at 273 nm, and the corresponding DSL photograph for the as‐synthesized CSS NPs dispersed in cyclohexane under laser irradiation at 273 nm with a power density of 10 mW cm^–2^. Reproduced with permission.[[qv: 20b]] Copyright 2016, Royal Society of Chemistry.

**Figure 11:**
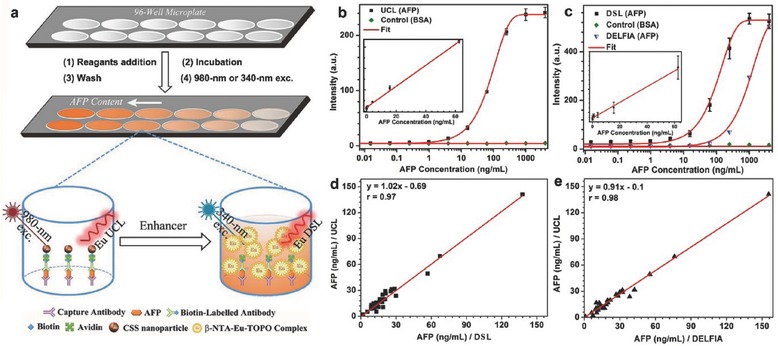
a) Schematic illustration of the proposed UCL and DELBA bioassays of AFP based on the UCL and dissolution‐enhanced PL of Eu^3+^ in the biotinylated CSS NPs. b) Calibration curves for AFP detection via UCL bioassay based on the biotinylated CSS NPs. c) Calibration curves for AFP detection via DLEBA based on the biotinylated CSS NPs and commercial DELFIA kit based on Eu^3+^‐DTTA complex, respectively. The control experiments in b) and c) were conducted by using BSA instead of the AFP antigen as analyte under otherwise identical conditions. By contrast, the UCL and TRPL signals were hardly detectable due to the absence of specific binding between BSA and anti‐AFP antibody. The insets of b) and c) show the corresponding linear range of the calibration curve for AFP detection via UCL and DELBA bioassays. d, e) Correlations between UCL bioassay, DELBA and commercial DELFIA kit for the AFP detection in 20 human serum samples, where the ordinate and abscissa denote the AFP level determined in UCL bioassay, DELBA and DELFIA kit, respectively. Reproduced with permission.[[qv: 20b]] Copyright 2016, Royal Society of Chemistry.

Very recently, based on a unique core/shell/shell (CSS) nanostructure design, we constructed Eu^3+^‐activated NaGdF_4_:Yb/Tm@NaGdF_4_:Eu@NaEuF_4_ NPs with the function of both efficient UCL and intense dissolution‐enhanced DSL of Eu^3+^ for the ultrasensitive detection of AFP.[[qv: 20b]] The spatial separation of Tm^3+^ and Eu^3+^ in the inner core/shell structure can effectively avoid the serious cross relaxation between them, thus enabling efficient UCL from both Tm^3+^ and Eu^3+^ under 980‐nm NIR laser excitation through Yb/Tm double sensitization or Gd‐mediated energy migration process (**Figure** **10**). The outer NaEuF_4_ shell can not only protect the UCL from surface quenching, but also serve as a source of Eu^3+^ to generate the intense dissolution‐enhanced PL in the enhancer solution. In a similar protocol to the assay of CEA, we constructed the sandwich‐type UCL and DELBA assays for AFP by the labeling of the biotinylated CSS nanoprobes. As a result, the concentration of AFP can be quantified by measuring the UCL signals of Eu^3+^ of the nanoprobes bound to a 96‐well microplate upon 980‐nm NIR excitation. After the UCL assay, the AFP concentration was further analyzed by measuring the dissolution‐enhanced TRPL signal of Eu^3+^ upon addition of the enhancer solution (**Figure** **11**a). Such a dual‐mode bioassay may act as a self‐referential validation for evaluating the accuracy and reliability of both assays (Figure 11d). From the calibrations curves (Figures 11b and 11c), the LODs were determined to be approximately 20 pg mL^–1^ (290 fM) for UCL bioassay and 60 pg mL^–1^ (870 fM) for DELBA, respectively, with their linear dynamic ranges of 0.01–60 ng mL^–1^ and 0.06–60 ng mL^–1^. To the best of our knowledge, the LOD of 20 pg mL^–1^ is the lowest among luminescent bioassays for AFP ever reported and is 30 times lower than that of commercial DELFIA kit (600 pg mL^–1^).^26^, ^44^ The slightly higher LOD of DELBA than that of UCL assay may be ascribed to the releasing of Gd^3+^ and Yb^3+^ ions from the inner shells of the CSS NPs, which compete with Eu^3+^ for the coordination with β‐NTA in the enhancer solution. Such low LODs coupled with the wide linear detection ranges of AFP in both UCL assay and DELBA are highly desirable to trace the serum AFP levels in the intervals for early diagnosis of HCC and monitoring HCC relapse after hepatectomy.

The AFP levels in 20 human serum samples determined independently by UCL assay, DELBA, and the commercial DELFIA kit were essentially consistent (Figures 11d and 11e), thus verifying the high reliability of the assays. The CVs for the assays of human sera by the addition of distinct amount of AFP standard solutions were below 5% and the analytical recoveries were in the range of 102–109% (Table 2), reconfirming the high accuracy and precision of the red luminescent CSS nanoprobes for AFP detection. Furthermore, these CSS nanoprobes were also explored for dual‐mode UCL/DSL bioimaging of human lung cancer cells (H1299), thus revealing the great potential of these dual‐mode CSS nanoprobes for cancer diagnosis both in vitro and in vivo.

## 5 Conclusions and Prospects

In conclusion, we have developed a novel luminescent bioassay method, namely, DELBA for the ultrasensitive in vitro detection of tumor markers, based on the intense dissolution‐enhanced PL of inorganic Ln^3+^‐nanoprobes in a PL enhancer solution. By replacing organic Ln^3+^‐chelates with inorganic Ln^3+^‐NPs in the protocol of commercial DELFIA, the labeling ratio of Ln^3+^ per biomolecule is significantly enhanced due to the highly concentrated Ln^3+^ in a single NP. Therefore, the PL signal of the system is enormously amplified, resulting in a much higher detection sensitivity than DELFIA. Based on the proposed DELBA, we have realized the ultrasensitive detection of CEA, PSA and AFP, with LODs several orders of magnitude improvement relative to those of either DELFIA or the assays utilizing the PL of the original Ln^3+^‐nanoprobes, which are hitherto the lowest values among luminescent bioassays reported previously.

However, there are still many challenges and economic concerns have to be overcome towards the commercialization of DELBA. First, an overall optimization of the nanoprobes should be carried out to guarantee the best performance of the assay, including the particle sizes, compositions, and surface functionalization of the NPs. In view of their superior dissolution ability in the acidic enhancer solution, Ln^3+^‐oxide NPs are considered to be ideal nanoprobe candidates for DELBA. Second, it is highly demanded to develop multiplex bioassay based on DELBA to increase the diagnostic rate of cancers and also for their high‐throughput screening. DELBA systems based on the chelating ligand of PTA that can simultaneously sensitize the PL of Eu^3+^, Sm^3+^, Tb^3+^ and Dy^3+^ may meet this requirement. Third, the cost of the reagents, instruments and labor involved in DELBA should be reduced, in order to promote the competitiveness of DELBA in the market of tumor marker detection. We believe that such a goal can be achieved through the overall optimization of the assays, for example, by simplifying the assay procedures or by combining DELBA with other state‐of‐the‐art testing devices such as lateral‐flow test strip and microfluidic chip. Last but not the least, as a merit of the ultrahigh sensitivity, the proposed DELBA are ideal for non‐invasive analysis of human glandular secretions like saliva and breath condensate, which remains unexplored and may revolutionize current serum‐based bioassays for future community or family medical practice. In this sense, the development of DELBA offers new opportunities for the early‐stage detection, monitoring or screening of cancer, thereby opening up new avenues for the high‐value utilization of rare earth.
